# Observations on an Occasional Increase and Decrease of Bulk in the Hair of the Head

**Published:** 1811-10

**Authors:** Thomas Forster


### *Observations on an occasional Increase and Decrease of Bulk in the Hair of the Head. By* THOMAS FORSTER, *Esq.*

(Nichols. Jour.)

THE sympathies between the skin and the stomach have been frequently adverted to by physiologists; the skin has been found to be alternately *hot and dry*, *hot and moist*, *cold and*

*and dry*, and *cold and moist;* and these varieties have been attributed to variations in the state of the stomach, between which and the skin a very direct sympathy is believed to exist. But the variations in the appearances of the hair do not appear to be duly noticed.

I have remarked, that people of what is usually called nervous and susceptible constitutions appear at times to have but half the quantity of hair on their heads, that they have at others, though they have assured me none had been cut or combed off. On minute examination I have found, that the apparent increase in quantity at certain times was occasioned by the following circumstances: the shafts themselves were found to be specifically larger, and more tense or elastic, at the same time that they did not lie in such close contact. The apparent diminution in quantity, at other times, I found to result from a specific decrease in the size of the shafts, which also lay in closer contact than ordinary, and were more flaccid, and generally more dry. Considering the considerable influence which the atmosphere exercises on our bodies, I was once induced to attribute the *closer contact* of the shafts to a diminution in their *electricity*, by which they would become less *mutually repulsive;* this however does not seem calculated to account for their increase in size. May the shaft be considered to be organized throughout, and its enlargement to be caused by an increased action of its vessels? or, Is there an aëriform perspiration into the cavity of the shaft, on an increase of which it becomes distended? or may the increased tension and size of the shaft be considered as resulting from the cooperation of these two causes?

The strength and tension of the hair appears generally to accompany health, while the weakness, close contact, and flaccidity of it denote disorder. I have observed also, that small doses of mercury have changed the appearance of the hair very soon after their administration. From being flaccid, dry, and small, it has become tense, strong, and moister; at the same time more tension and solidity has appeared in the muscles, and the countenance has displayed a more healthy appearance. Now mercury may increase an aëriform perspiration, (if such a one exist) into the shaft; it may also set the digestive organs to rights, thereby cause a more healthy action of the vessels in general, and of those of the shaft among the rest. I cannot help observing, that there is no objection to supposing hairs organized, because we cannot discover their vessels. On this subject we may, I think, be allowed to reason thus: If all nourishment be performed by the action of vessels, either vascularity must extend itself *ad infinitum*, or there must be certain small vessels not nourished rished at all. Can we demonstrate those small arteries, which ramify in the coats of and nourish the smallest *vasa vasorum*? Such considerations as these ought to prevent our denying organization to any part of an animal body, event o the cuticle and the enamel of the teeth.

